# Presenting new stimuli to study emotion: Development and validation of the Objects-on-Hands Picture Database

**DOI:** 10.1371/journal.pone.0219615

**Published:** 2019-07-24

**Authors:** Natália Lisandra Fernandes, Josefa N. S. Pandeirada, James S. Nairne

**Affiliations:** 1 CINTESIS, UA, University of Aveiro, Aveiro, Portugal; 2 William James Center for Research, University of Aveiro, Aveiro, Portugal; 3 Purdue University, West Lafayette, IN, United States of America; Victoria University of Wellington, NEW ZEALAND

## Abstract

A long-standing goal shared by researchers has been to design optimal experimental procedures, including the selection of appropriate stimuli. Pictures are commonly used in different research fields. However, until recently, researchers have relied mostly on line-drawings, which can have poor ecological validity. We developed a set of high quality standardized photographs of objects from six different categories, recorded under two camera viewpoints, and five presentation conditions (on its own, held by clean hands, and by hands covered with different substances: sauce, chocolate and mud). These various staging conditions can be used to induce different emotional states while maintaining the object of interest constant. We first report normative data on the objects’ name agreement and familiarity collected from North American and Portuguese participants. Results showed high name agreement and familiarity in both samples. Next, arousal, disgust and valence ratings were collected for the stimuli under either an emotional-activating or a neutral context. Subjective ratings varied according to the staging condition and the context, confirming that the same items can effectively be used in different emotional conditions. This database allows researchers to select more ecologically-valid stimuli according to their research purposes while considering several variables of interest and avoiding item-selection problems commonly present when comparing responses to neutral and emotional items.

## Introduction

Images of everyday objects have been used as stimuli in a wide array of research fields, such as perception (e.g., [1]), attention (e.g., [2]), memory (e.g., [3]), language (e.g., [4]), and neuroscience (e.g., [5]). Over the last few decades, several sets of pictures have been created and standardized, allowing researchers to select the most suitable stimuli for their specific research needs. Snodgrass and Vanderwart [6] developed one of the most influential and widely used set of visual stimuli– 260 black-and-white line drawings depicting mostly objects. This database has been progressively expanded and enriched with the addition of new pictures (e.g., [7, 8–10]). Additionally, it has been validated and standardized for different cultures, languages and age groups (e.g., [11, 12–14]). However, an increasing number of researchers have started to use photographs as their stimuli, which provide a more ecological and realistic representation of objects (e.g., [15, 16–18]).

Line-drawings and photographs are characterized by different features, which differently affect the processing of stimuli. For example, they seem to recruit different semantic processes, which potentially impacts how the object is attended to, named and recognized [19, 20]. Whereas line-drawings are simple prototypical schematic representations of objects, photographs are a more realistic depiction of objects, containing richer surface information such as color, texture, shadow, and occasionally background details [16]. Such stimuli are particularly useful for researchers concerned with the ecological validity of their procedures, and who wish to create experimental conditions that more closely mimic real-life situations. Indeed, researchers have suggested that “using photos as the experimental stimuli increases the chances of activating the same neuronal circuits that are activated in daily tasks” ([19], p. 2).

Despite their widespread need and broad potential use, few databases of photographs are available for researchers to use. One of the fields in which images have been increasingly and commonly used is emotion induction. Some sets of standardized emotional stimuli are available and have been frequently employed, for example, to analyze the impact of emotions on cognitive processes as well as to gain a better understanding of the dynamics and underlying mechanisms of emotional processes [21]. The International Affective Picture System (IAPS; [22]) is the most used picture database, although there has been an increasing interest in the development of other emotionally evocative databases aiming to overcome some of the limitations of the IAPS (e.g., the Geneva Affective Picture Database: [21], the DIsgust-RelaTed-Images: [23], the Nencki Affective Picture System: [24], the Set of Fear Inducing Pictures: [25], the Emotional Picture System: [26]). However, these databases only allow researchers to compare the processes that occur during the exposure to different items (e.g., disgusting vs. non-disgusting items) introducing potential item-selection concerns–that is, inherent and potentially uncontrolled item properties that could impact the processes under scrutiny. We reasoned that the best way to solve this problem would be to develop a database containing exactly the same stimuli (objects) but recorded under conditions in which the objects can be processed in domain-specific ways (e.g., as disgusting, neutral or fear-evoking). For this to be possible, one needs a dataset of stimuli that could be effectively encoded in different conditions; for example, the sauce covering the hands holding an object could be described as being pasta sauce or vomit; such different descriptors should activate low vs. high disgust and be weakly vs. strongly arousing, respectively. Thus, even though we would be able to manipulate the emotion being activated by the picture, the object of interest would be the same in the two conditions. This effectively eliminates item-selection concerns that arise when, for example, responses or processes to disgusting and non-disgusting stimuli (which might perceptually differ in many ways) are directly compared.

The law of contagion–a specific kind of sympathetic magical thinking that entails the belief that properties can be transferred through direct physical contact [27–29]–can inspire many research avenues using this type of stimuli. For example, researchers could use exactly the same objects to study contamination by simply altering the description of the hands holding those objects. Following this idea, and with the goal of providing stimuli that would allow researchers to use them in multiple contexts, we created a database of photographs of different objects being held by hands under four different conditions: clean hands, hands covered with mud, hands covered with pasta sauce, and hands covered with chocolate and peanut butter spread. Each object was also photographed on its own (without the hands) sitting on a table. Even though some researchers have recently proposed alternative sets of photo stimuli (e.g., [16, 19, 23, 24]), to the best of our knowledge, none provides photographs of exactly the same stimuli (objects) recorded under presentation conditions that are capable of inducing different emotional states. A database containing pictures of the exact same stimuli (objects) in contact with different substances will open new opportunities for researchers interested in exploring, for example, the law of contagion, emotional processes, as well as other phenomena. Furthermore, by virtue of being held by hands, our stimuli convey a social dimension that does not exist in other databases which, for the most part, present the objects on their own.

The current study presents a new database of stimuli containing photographs of 126 everyday objects from six different categories: women’s accessories, fruits, kitchen utensils, office supplies, toys, and vegetables. These comprise the Objects-on-Hands Picture Database. The photographs were taken in a highly controlled environment and under two different viewpoints (frontal and top viewpoints). These two camera viewpoints could allow different conceptualizations of the object with respect to the participant. For example, the top viewpoint could represent the object as if it were being held by the participant, whereas the frontal viewpoint could be described as someone else approaching the object; these different perspectives could also be conceptualized as corresponding to the participant giving or receiving the object, respectively. Therefore, these alternative perspectives of the same object afford a set of interesting experiments in the domain of social interaction.

Furthermore, our stimuli include objects from different categories which can be used to explore organizational or relational aspects of the processing and/or effects. For example, some research has explored whether the mnemonic advantage for emotionally-valenced material, as compared to neutral material, is related to a stronger organizational structure of the former as compared to the later (e.g., [30]). Once again, such studies compared different stimuli and, therefore, their results are fraught with potential item confounding. With our database, such a problem could be avoided while exploring the variable of real interest: the relational processing that could be naturally afforded by using exactly the same category items but under different emotionally-activating contexts.

The Objects-on-Hands Picture Database can also be of use in several applied research areas and across tasks involving perception, attention, memory, and behavioral experiments. For example, contagion beliefs have been found across a variety of contexts (e.g., marketplace, workplace, etc.) and have proven to be highly influential in guiding cognitive and behavioral processes. In this sense, people evaluate more negatively and are unwilling to interact with objects that have been in close contact with disgusting stimuli (e.g., [31, 32,33]). The law of contagion has inspired evolutionary researchers to study disease-avoidance strategies designed by natural selection to cope with life-threatening pathogens. For example, selection pressures imposed by pathogens are thought to have driven the evolution of the emotion of disgust, which motivates the avoidance of disease-causing microorganisms [34]. Memory seems to be biased or tuned to remember disgusting and disease-related information [35]. These are just some of the key components that have been proposed to embody a “behavioral immune system”, a system that has concomitantly evolved with the “biological immune system” to protect us against pathogen threat [36]. Our stimuli can be used to further explore the operating mechanisms and the bidirectional relationship of these two systems.

The database can also be used in clinical research. For example, our stimuli could be used to explore responses to neutral or disgust-evoking stimuli in subjects with multiple forms of maladaptive behaviors and psychopathology as well as to investigate their role in the etiology, maintenance, or treatment of such conditions (e.g., substance abuse and drug addiction: [37], eating disorders: [38], obsessive-compulsive disorder: [39], among others). Likewise, our dataset can be used to study topics as diverse as the desirability of stimuli presented in different conditions (e.g., food items when held by hands covered with chocolate vs. by clean hands) depending on factors such as mood (e.g., [40]). In the social area, one could explore the willingness of participants to accept a given object, or the value assigned to it, when it is offered by people described with different personal characteristics. These characteristics, in turn, could be associated with more positive or negative social stereotypes [32]. By presenting the objects held by real hands potentially increases the influence of such stereotypes. Furthermore, being able to present the objects from different perspectives–from the giver’s perspective (top viewpoint) or from the receiver’s perspective (front viewpoint) adds other investigation opportunities. Therefore, the potential usage of this new database is as large as the numerous areas in which emotion has been shown to affect cognition and behavior.

Language and cultural differences in picture naming and rating tasks (e.g., [14]) have long been acknowledged, highlighting the need for researchers to adjust the selection of stimuli as a function of the cultural background of the participants. Additionally, name agreement and familiarity are known to affect performance in different areas, such as language, perception, and memory (e.g., [41, 42, 43]). Therefore, besides creating the pictures database we also collected information on the naming and familiarity of the stimuli. This was done with a group of North American participants and a group of Portuguese participants. These data will allow researchers to make sure that they are selecting stimuli that are identifiable and familiar to their participants. To this end, the frontal-view photographs of the objects being held by clean hands were presented individually and participants were asked to provide a name for the object and then to rate how familiar they were with it. A comparison between groups (North American vs. Portuguese) on name agreement and familiarity ratings is presented to explore the viability of using these stimuli in cross-cultural studies.

Additionally, we aimed to demonstrate that different contextualizations of the stimuli can activate different emotional states, such as arousal, disgust and valence. To this end, the frontal-view photographs of the objects being held by hands covered with chocolate were rated on these dimensions but, in different groups, the images were framed in a disease context, a non-disease context, or presented with no context. We expected to obtain different evaluations of the same stimuli according to the provided contextualization. Such results would demonstrate that researchers can safely use the exact same stimuli in their research while, at the same time, elicit different emotional reactions.

In sum, this study aimed to create a dataset of photographs of real objects belonging to six different categories under different viewpoints and conditions; such a variety will allow a large possibility of experimental manipulations in different research contexts (Phase 1: Development of the Database). Additionally, we provide normative data (Phase 2: Validation of the Database) on the objects’ name agreement and familiarity in two different countries (study 1), as well as ratings of arousal, disgust and valence of the same stimuli but presented in different context conditions (study 2). All procedures have been approved by the Ethical council at the University of Aveiro and by the IRB from Purdue University; they are all in accordance with the 1964 Helsinki declaration and its later amendments.

## Phase 1: Development of the database

### Method

#### Objects selection

We selected approximately 20 objects from each of six different categories: fruits (e.g., pear; *N* = 20 images), vegetables (e.g., onion; *N* = 21 images), kitchen utensils (e.g., bowl; *N* = 19 images), office supplies (e.g., tape; *N* = 21 images), toys (e.g., dices; *N* = 23 images), and women’s accessories (e.g., lipstick; *N* = 22 images). These were selected considering that the objects could easily be held by hands while also being easily identifiable. Category norms for 45.24% of these stimuli can be found in [44]; these could be of interest for researchers interested in taking into account categorization information.

#### Conditions

Each stimulus was photographed under five different conditions. In four of the conditions, stimuli were held by hands with different presentations: clean hands (clean condition), hands covered with mud (mud condition), with a pasta sauce (sauce condition), or with a mixture of chocolate and peanut butter spread (chocolate condition). The latter three conditions were created as they afford the processing of different contexts. For example, images in the sauce condition can be associated with a disease context (covering described as vomit) or with a cooking situation (covering described as pasta sauce), in order to induce different affective states. In a fifth condition, objects were photographed on a table covered with a white sheet (object condition). These last photographs can be used, for example, in a final recognition test for the objects irrespectively of the condition employed at encoding. The hands holding the stimuli across all of these conditions were the same.

#### Image acquisition and processing

**Fig 1:**
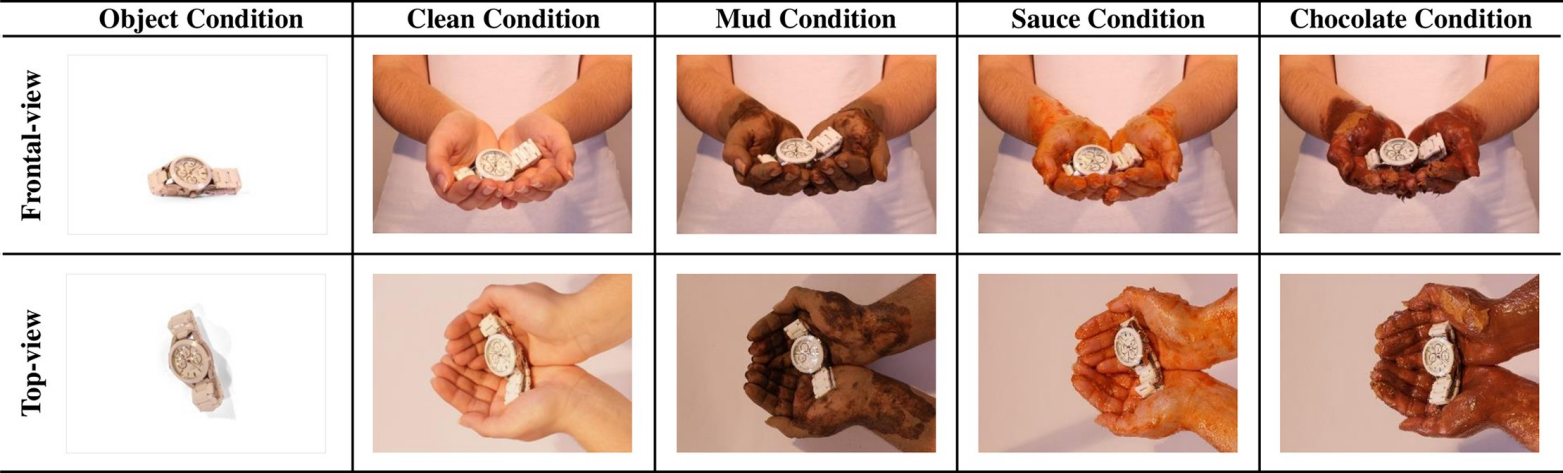
Examples of stimuli in each condition and camera viewpoints.

Images were recorded in a controlled environment in the audio-visual studio at the Department of Communication and Art at the University of Aveiro (Portugal). High-quality digital color photographs of 126 objects were taken against a uniform white background from two different camera viewpoints (i.e., frontal and top viewpoints). Care was taken to position the stimuli in a similar way across all of the conditions in which the object was being held by hands. We also tried to maintain the same position, as best as possible, when the objects were placed on the table. Fig 1 displays examples of pictures in each of the conditions and camera viewpoints. A total of 1260 images were collected; however, due to technical reasons (i.e., problems during the transference process from the cameras to the computer), we only have 1171 images available (see S1 Appendix for information of the stimuli available in each condition, available at https://osf.io/xn2u9/). Because neither the objects nor the necessary photo shooting conditions were met when we noted this problem, we were not able to recollect the missing stimuli. The database is freely accessible via a web-interface at https://sites.google.com/view/adaptive-memory-lab/data-databases.

The frontal-view photographs were captured with a Canon EOS 70D camera with a resolution of 5472 x 3648 pixels (CMOS sensor of 20 megapixels) in 22.5-bit color RAW format. The camera was mounted on a tripod placed at a distance of 50 cm from the stimuli. Height of the camera was 88 cm for stimuli being held by hands and 79 cm for stimuli placed on the table. Top-view photographs were acquired using a Canon EOS 60D camera with a resolution of 5184 x 2916 pixels (CMOS sensor of 18 megapixels) in 24-bit color RAW format. The camera was positioned above the stimuli on a tripod with a height of 160 cm and a distance to the object of approximately 72 cm. For both cameras focal length and focus distance was set to 50 mm. The sensor light sensitivity (ISO) was fixed at 800. An exposure time of 1/60 sec and take aperture of ƒ/20 were used.

**Fig 2:**
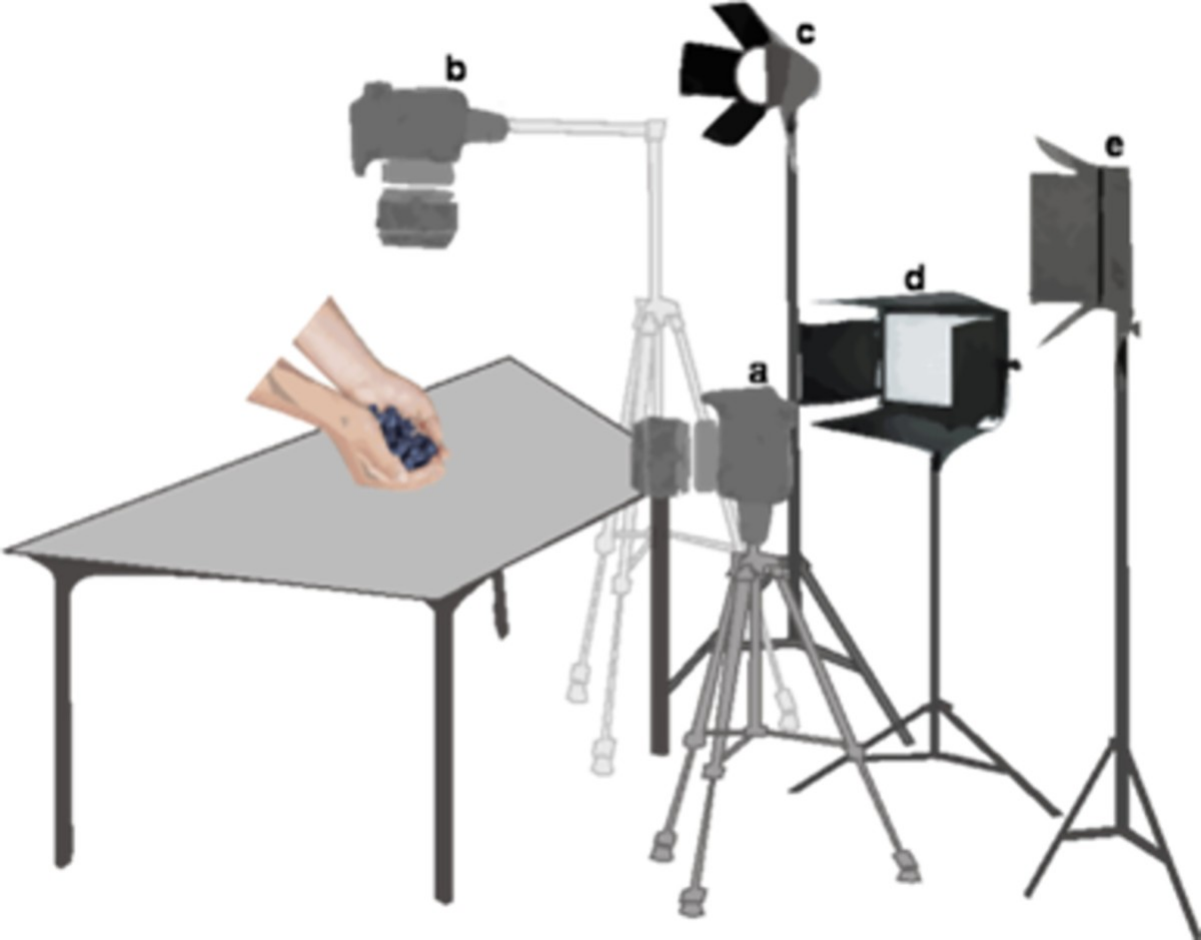
Spatial arrangement of the equipment used to acquire the images. (a) frontal-view camera, (b) top-view camera, (c) halogen spotlight (a similar spotlight was positioned on the left side of the frontal-view camera but is not here represented), (d and e) LED spotlights (a spotlight similar to d was positioned on the left side of the frontal-view camera but is not here represented).

Three white light-emitting diode (LED) spotlights (16x16) with a color temperature of 7000 kelvin, and two 1250-watt halogen spotlights were used to obtain optimal lighting conditions. One of the LED spotlights was located at a height of 155 cm and about 73 cm behind the frontal-view camera. The remaining two were located at a height of 110 cm, one at 60 cm on the right side and the other at 60 cm on the left side of the frontal-view camera. The halogen spotlights were positioned at a height of 164 cm and 57 cm from the frontal-view camera, one on the right and one on the left side (see Fig 2 for a schematic presentation of the setting). Frontal-view images were spatially aligned and manipulated using *Adobe Photoshop CC* so that the position of the body, hands and stimuli were as similar as possible in all conditions and for all stimuli. Furthermore, the stimuli photographed on the table were edited to remove the background of the images; thus, each photograph included the object’s image only (see Fig 1).

## Phase 2: Validation of the database

### Study 1: Name agreement and familiarity

Name agreement information and familiarity ratings (on a scale of 1–5) were collected for each of the 126 frontal-view stimuli from the clean condition from two different cultural groups: North Americans and Portuguese. Data are first presented separately for each group and then comparisons between groups were conducted. To further explore if the data collected in this presentation condition would apply for the remaining presentation conditions, we asked another Portuguese sample of participants to provide the name and familiarity ratings for the objects presented in the sauce condition (frontal-view perspective). The comparison of the data obtained in the two conditions (clean and sauce) provides preliminary evidence that the normative information can be applied to the other photo settings.

## Method

### Participants

The North American sample included seventy-eight psychology students from Purdue University (women = 25; 32.05%), aged between 18 and 36 years old (*M*_*age*_ = 19.65 years, *SD* = 2.37), who participated in exchange for course credits. All participants were native English-speakers (data from an additional 11 non-native English speakers were excluded). The Portuguese sample included 293 native European Portuguese speakers (women = 200; 68.03%), aged between 18 and 70 years old (*M*_*age*_ = 36.11 years, *SD* = 13.33). One hundred and seventy one participants (*M*_*age*_ = 36.86 years, *SD* = 14.16) provided the names and familiarity ratings for the objects in the clean condition, and 122 participants (*M*_*age*_ = 35.07 years, *SD* = 12.10) provided this same information for the objects in the sauce condition (data from an additional 3 non-native Portuguese speakers were excluded). The Portuguese sample participated voluntarily and no compensation was offered. Informed consent was obtained from all participants before beginning participation.

### Materials

In this task, the 126 frontal-view stimuli from the clean condition were used. However, each participant only responded to a sub-group of 63 stimuli previously created in a pseudo-random manner from the initial pool of stimuli; these subgroups were created in order to avoid a lengthy questionnaire and to prevent abandonment of the task. Care was taken to ensure that each sub-group of stimuli included a similar number of objects from each of the six categories. Order of presentation of the stimuli was randomly determined for each participant. The corresponding 126 frontal-view stimuli from the sauce condition were also used and presented in these same conditions.

### Procedure

#### North American sample

The experiment was prepared using the *Qualtrics survey software* (Qualtrics Labs Inc., Provo, UT). The experiment was run in groups of up to four participants but each participant responded to the task in an individual workstation. This task was performed after they responded to other non-related tasks.

Objects were presented one at a time on the computer screen along with the naming question and the familiarity rating task. For the naming task, participants were asked to identify the object presented in the picture and to type the first name that came to their mind into a dialog box. When participants could not provide the name of the stimuli, they were asked to indicate whether this was because: (1) they did not recognize the object; (2) they knew the object but not its name; or, (3) they knew the name but were unable to retrieve it at that moment. These responses were provided by selecting the options “don’t know object” (DKO), “don’t know name” (DKN), or “tip-of-the tongue” (TOT), respectively. The familiarity rating task was displayed below the naming question; here, participants were asked to indicate the level of familiarity they had with the object. Responses were provided using a 5-point Likert scale (1 = very unfamiliar, 2 = somewhat unfamiliar, 3 = neither familiar nor unfamiliar, 4 = somewhat familiar, and 5 = very familiar) by clicking on the response of their choice with the computer mouse. The tasks were self-paced but participants were instructed to respond quickly and to rely on their “gut instinct”. After responding to the naming and familiarity rating tasks participants hit a “next” button which led to the presentation of the next stimuli. The questionnaire ended with the collection of information regarding sex, age, and nationality. The task lasted approximately 10 minutes.

#### Portuguese sample

Data was collected via the World Wide Web using the *Qualtrics survey software* (Qualtrics Labs Inc., Provo, UT). A brief description of the study along with the electronic link to access the questionnaire was sent by electronic mail to Portuguese public and private universities for dissemination. The opening page of the questionnaire provided a brief description of the study along with confidentiality information and an informed consent request. If no consent was granted, participants were thanked for their interest and the program ended; otherwise, the program moved on to the initial instructions. The procedure was as described for the North American sample but with all instructions presented in European Portuguese. No indication was given about the substance that was covering the dirty hands (sauce).

#### Data analysis

For each object we report the modal name, name agreement, nonmodal names and naming failures as defined next; the familiarity ratings are also provided. Some corrections to the individual entries were conducted by the first author before determining these variables. For example, misspelled names were rewritten in their orthographically correct form (e.g., from “cantelope” or “canteloup” to “cantaloupe”). When two or more names were provided by the participant to the same object, only the first one was included in the data analysis.

#### Modal name

This corresponds to the name assigned to each stimulus by the highest percentage of participants within each sample. Composite names (e.g., “garlic clove” and “clove of garlic”) or specifications/ adjectives presented along with the object name (e.g., “apple” and “green apple”) were considered as corresponding to the same name; among these, the most frequent name was considered the modal name. For example, for the image of “gloves” the modal name was “gloves” (*n* = 41), but the responses “winter gloves” (*n* = 1) and “women’s gloves” (*n* = 1) were considered in the total frequency of the modal name (final frequency *N* = 43).

#### Name agreement

Refers to the degree of agreement among participants on a specific modal name. Two measures of name agreement were computed: (1) the percentage of participants naming the stimuli with its modal name (%NA), and (2) the *H* statistic (Shannon, 1949), which measures the variability of answers across participants. The *H* value was computed using the following formula developed by Snodgrass and Vanderwart [6]:

$$h=\sum_{i=1}^{k}p_{i}log_{2}(\frac{1}{p_{i}})$$

where *k* refers to the number of different names given to each image and *p_i_* indicates the proportion of participants giving each name. Naming failures (DKO, DKN, and TOT responses) were not taken into account when computing the *H* values (for more information see [6]). The %NA provides important information about which items elicit the same response from all participants and which items lead to more naming failures, whereas the *H* statistic is a more reliable measure of the distribution of names across participants. For example, “if two concepts both are given their dominant name by 60% of the subjects, but one is given a single other name and the second is given four other names, both concepts will have equal percentage agreement scores, but the first will have a lower H value” ([6], p. 184). Thus, higher %NA values signify greater name agreement; in turn, higher *H* values indicate lower levels of name agreement derived from a higher variability in names given by participants. Therefore, a negative correlation is expected between these two measures. An item is given an *H* score of 0 when the same modal name is provided by all participants with a valid response (i.e., excluding naming failures), and a %NA score of 100% (i.e., perfect name agreement) when the modal name is provided by the entire sample.

#### Nonmodal names and naming failures

Includes the names that differed from the modal name and that were considered alternative names or nonmodal names. The nonmodal names were categorized as correct or incorrect. Correct nonmodal names refer to other ways of appropriately identifying the stimuli presented (e.g., synonyms), or to more general or specific designations of the modal name (e.g., semantic category of the object). Nonmodal names were considered incorrect when they referred to a stimulus that does not match to the one depicted in the image. Specific examples of these cases are provided in the Results section. Name frequency for each nonmodal name was calculated and converted to percentages. Frequency and percentage of naming failures (DKO, DKN, and TOT responses) were also calculated for each image.

#### Familiarity

Refers to the degree to which the stimulus is familiar to participants. Familiarity was computed by averaging the scores reported on the 5-points Likert scale; standard deviations are also presented. Similar to the results obtained in previous studies (e.g., [16, 45]), we expected familiarity to correlate positively with %NA and negatively with the H score.

#### Statistical analysis

Analyses were conducted on the data by stimuli. Correlations between the two name agreement measures (%NA and H), as well as between these measures and familiarity, were evaluated using Spearman's correlation coefficient. The results obtained from the North American and Portuguese sample were compared using mixed ANOVAs where the sample was considered a within-subject variable and object category was considered as a between-subjects variable. Whenever significant interactions occurred these were followed by further analysis (e.g., comparison among categories within each sample) that assumed the necessary corrections for multiple comparisons (e.g., Gabriel's post-hoc tests). This was done on the variables related to name agreement (%NA and H), naming failures (DKO, DKN and TOT), and familiarity. To compare these same results obtained for the same objects when these were held by clean hands or by the hands covered with sauce, we also conducted mixed ANOVAs with stimulus condition as a within-subject variable and category as a between-subjects variable. The same post-hoc analyses as before were conducted to clarify interactions. The level of statistical significance was set at .05 (two-tailed). All analyses were performed using the Statistical Package for Social Sciences (IBM SPSS) version 24.

## Results and discussion

The indexes obtained for each stimulus and in each sample are presented in an Excel file made available as S2 Appendix (available at https://osf.io/xn2u9/). For each stimulus, the responses coded as modal names, the different alternative names provided, along with their corresponding frequencies and percentages of occurrence, are listed in S3 Appendix (available at https://osf.io/xn2u9/). We start by presenting the data on name agreement and familiarity collected for the stimuli from the clean condition in both the North American and the Portuguese samples; data from these two samples were then compared. The same data are then reported for the sauce-condition stimuli followed by their comparison with the data from the clean condition (both obtained from independent Portuguese samples).

### Data from the North American sample (clean condition)

Each of the 126 stimuli was rated approximately by 42 (*SD* = 1.6) native English participants. The average percentage of participants naming the stimuli with its modal name (%NA) was 75.1% (*SD* = 27.3%; negatively skewed, with a range from 6.8% to 100%). The *H* statistic was 0.66 (*SD* = 0.75; positively skewed, with a range from 0 to 2.97), indicating relatively high name agreement (see Table 1; for an in-depth analysis see Tables 2 and 3). As expected, the two measures of name agreement (%NA and *H*) showed a high negative correlation, *r*_*s*_ = -.923, *p* < .001.

**Table 1:** Summary statistics for the name agreement scores (%NA and *H* values) and familiarity ratings, given by the North American and the two Portuguese samples.

	%NA			H			Familiarity		
	American^a^	Port.^a^	Port.^b^	American^a^	Port.^a^	Port.^b^	American^a^	Port.^a^	Port.^b^
Mean	75.1%	84.4%	84.3%	0.66	0.51	0.50	4.20	4.54	4.49
Std. Dev.	27.3%	17.8%	17.9%	0.75	0.58	0.58	0.72	0.37	0.37
Median	86.4%	92.7%	92.5%	0.34	0.23	0.25	4.35	4.61	4.55
Q1	54.3%	71.8%	73.3%	0.00	0.00	0.00	3.94	4.34	4.29
Q3	100.0%	98.8%	98.4%	1.12	0.91	0.87	4.76	4.83	4.80
IQR	45.7%	27.0%	25.1%	1.12	0.91	0.87	0.82	0.49	0.51
Min	6.8%	32.1%	33.3%	0.00	0.00	0.00	1.65	3.33	3.17
Max	100.0%	100.0%	100.0%	2.97	2.07	2.22	5.00	5.00	4.98
Skewness	-0.87	-1.09	-1.08	1.08	1.04	1.13	-1.38	-1.23	-0.98

American = North American sample; Port. = Portuguese sample

a = clean condition

b = sauce condition

Std. Dev. = standard deviation; Q1 = 25th percentile; Q3 = 75th percentile [SPSS computation of percentiles: (w+1)*p (w is the weighted case count)]; IQR = interquartile range (Q3-Q1); Min = minimum; Max = maximum.

**Table 2:** Proportion of stimuli with %NA equal to 100%, between 100% and 80%, between 80% and 50%, and below 50%, in each sample.

	American^a^	Port.^a^	Port.^b^
**100%**	25.4%	19.8%	22.2%
**< 100% and ≥ 80%**	32.5%	49.2%	46.8%
**< 80% and ≥ 50%**	20.6%	25.4%	26.2%
**< 50%**	21.4%	5.6%	4.8%

American = North American sample; Port. = Portuguese sample

a = clean condition

b = sauce condition

**Table 3:** Proportion of stimuli that yielded one, two, three, four, five, or more than five names in each sample.

	American^a^	Port.^a^	Port.^b^
**Single name (H = 0)**	32.5%	25.4%	31.7%
**Two names**	18.3%	26.2%	26.2%
**Three names**	17.5%	16.7%	11.9%
**Four names**	10.3%	10.3%	11.9%
**Five names**	9.5%	6.3%	7.1%
**More than five names**	11.9%	15.1%	11.1%

American = North American sample; Port. = Portuguese sample

a = clean condition

b = sauce condition

Five (3.97%) of the obtained modal names corresponded to misidentifications of the stimuli; for example, the vegetable “chayote” was not recognized by most participants (84.1% naming failures), with the remaining participants providing an inaccurate modal name (i.e., “green pepper”) or other unrelated names. Naming failures represent 13.1% of the data with a higher percentage of DKO responses, followed by the DKN and then the TOT (see Table 4). From those participants who gave a name (i.e., excluding naming failures), 83.7% provided the modal name, 7.0% provided a correct nonmodal name and 9.3% gave an incorrect nonmodal name. The majority of the correct nonmodal names were synonyms of the modal name (e.g., “scrunchies” for the modal name “hair ties”), more general (e.g., “melon” for the modal name “cantaloupe”) or specific (e.g., “toy airplane” for the modal name “toy plane”) designations of the modal name. On the other hand, some of the incorrect nonmodal names suggest that participants did not recognize the stimulus (e.g., providing the name “onion” for the picture of a “garlic”). Some images, however, elicited names of visually and/or semantically similar stimuli (e.g., providing the name “zucchini” for the photography of a “cucumber”), suggesting that those photos might not depict very clearly the object they intended to present.

**Table 4:** Proportion of naming failures (DKO, DKN and TOT responses), in each sample.

	American^a^	Port.^a^	Port.^b^
**% DKO**	7.8 (14.9)	1.1 (2.8)	1.3 (3.2)
**% DKN**	3.8 (6.0)	2.0 (3.8)	1.8 (3.2)
**% TOT**	1.5 (3.1)	1.6 (2.8)	1.3 (2.4)

American = North American sample; Port. = Portuguese sample

a = clean condition

b = sauce condition

DKO = don’t know object; DKN = don’t know name; TOT = tip-of-the tongue

Participants reported a relatively high degree of familiarity with the stimuli presented (*M* = 4.20, *SD* = 0.71; scale: 1–5). Familiarity showed a negatively skewed distribution, reflecting the fact that few stimuli were rated as being low on this dimension (see Table 1). Familiarity was strongly and positively correlated with %NA (*r*_*s*_ = .812, *p* < .001), indicating that more familiar stimuli elicited a higher name agreement. On the other hand, familiarity was negatively correlated with the *H* value (*r*_*s*_ = -.681, *p* < .001), suggesting that participants assigned more alternative names to less familiar objects.

### Data from the Portuguese sample (clean condition)

Each of the 126 stimuli was, on average, rated by 85.3 (*SD* = 3.5) native Portuguese participants. Results showed an overall high level of name agreement with 84.4% (*SD* = 17.8%) of the participants producing the modal name (range: 32.1% - 100%), and an *H* statistic of 0.51 (*SD* = 0.58; range: 0–2.07); See Table 1 for more descriptive results and Tables 2 and 3 for an in-depth analysis. As with the North American sample, the two measures of name agreement (%NA and *H*) were found to be strongly negatively correlated, *r*_*s*_ = -.939, *p* < .001.

All modal names were appropriate designations of the presented objects. The percentage of naming failures was 4.7%, with most referring to DKN, followed by TOT and finally by the DKO (see Table 4). A total of 88.2% of the participants who named the stimuli (i.e., excluding naming failures) provided the modal name, 7.8% provided a correct nonmodal name and only 4.0% gave an incorrect nonmodal name. Again, the majority of the correct nonmodal names were synonyms of the modal name (e.g., the names “aguça”, “afiadeira” and “apara-lápis” are regarded as synonyms of the name “afia” [“pencil sharpener”]). Correct nonmodal names can also be partly accounted for by Portuguese regional dialect variations (e.g., a bowl is called “tigela” in most of the country but is commonly known as “malga” in the northern part of Portugal) or by more general or specific designations (e.g., “esferográfica” [“ballpoint pen”] is a specific type of “caneta” [“pen”; modal name]). Incorrect nonmodal names suggest either that participants did not recognize the stimuli (e.g., the name “pepino” [“cucumber”] given to the picture of a “pimento” [“pepper”]) or were confused about what it represented (e.g., providing the name “abrunho” [“sloe”] instead of “uvas” [“grapes”]).

Participants' ratings of familiarity were negatively skewed, indicating that participants were in general very familiar with the stimuli (*M* = 4.54, *SD* = 0.37; scale: 1–5). As happened in the data from the North American sample, a significant positive correlation with the %NA (*r*_*s*_ = .595, *p* < .001), and a significant negative correlation with the *H* value (*r*_*s*_ = -.492, *p* < .001) was also found in this Portuguese sample.

### Comparing the North American and the Portuguese data (clean condition)

This section reports the comparison between the data obtained in the North American and the Portuguese sample regarding the clean condition stimuli. The former sample was less accurate in naming the stimuli, as denoted by a lower percentage of participants providing the modal name (%NA) and a higher mean *H* value, as compared to the Portuguese sample (see Table 1). In fact, a mixed ANOVA with sample and object category as factors revealed a significant main effect of sample for the %NA and the *H* value, *F*(1, 120) = 17.45, *MSE* = 0.56, *p* < .001, η_*p*_^2^ = .127, and *F*(1, 120) = 6.34, *MSE* = 1.62, *p* = .013, η_*p*_^2^ = .050, respectively; these data denote lower name agreement obtained in the North American sample as compared to the Portuguese sample. There were no significant effects of category for the %NA nor for the *H* value, *F*(5, 120) = 1.60, *p* = .164, and *F*(5, 120) = 1.97, *p* = .088, respectively. The interactions for the %NA and for the H value were also not statistically significant, *F*(5, 120) = 1.96, *p* = .090, and *F*(5, 120) = 1.64, *p* = .156, respectively.

There was a significant difference among categories on the DKN, *F*(5, 120) = 2.31, *MSE* = 0.01, *p* = .048, η_*p*_^2^ = .088 (the post-hoc analysis revealed no significant differences among categories), but not on the DKO and TOT, *F*(5, 120) = 1.01, *p* = .418 and *F*(5, 120) = 1.73, *p* = .133, respectively. The Sample x Category interaction was not significant for all of the naming failures (DKO: *F*(5, 120) = 1.72, *p* = .135; DKN: *F*(5, 120) < 1; TOT: *F*(5, 120) = 2.27, *p* = .052). However, as indicated by a significant main effect of sample, the North American participants committed more naming failures than the Portuguese participants, particularly with DKO and DKN responses, *F*(1, 120) = 30.73, *MSE* = 0.29, *p* < .001, η_*p*_^2^ = .204, and *F*(1, 120) = 11.91, *MSE* = 0.02, *p* = .001, η_*p*_^2^ = .090, respectively. No main effect of sample was found for TOT responses, *F*(1, 120) < 1. This higher proportion of naming failures from the North American sample could be due to the fact that the database was developed in Portugal containing objects that are common in Portugal but some that are somewhat uncommon to the North American participants (e.g., chayote, passion fruit, loquat).

Supporting this idea is the fact that familiarity ratings were significantly lower in the North American than in the Portuguese sample, as denoted by a significant main effect of sample, *F*(1, 120) = 51.75, *MSE* = 7.57, *p* < .001, η_*p*_^2^ = .301. There was no main effect of category, *F*(5, 120) = 1.23, *p* = .300, but the Sample x Category interaction was reliable, *F*(5, 120) = 4.63, *MSE* = 0.68, *p* = .001, η_*p*_^2^ = .162. North American participants, compared to the Portuguese ones, were less familiar with objects from all categories, except for those belonging to the category of toys, for which the familiarity ratings were similar to the ones reported by the Portuguese sample. Additionally, when we compared the familiarity ratings among categories in each sample, no significant effect was obtained in the North American sample, *F*(5, 120) = 1.40, *p* = .230, but it was reliable on the Portuguese data, *F*(5,120) = 4.63, *MSE* = 0.56, *p* = .001, η_*p*_^2^ = .162. Gabriel's post-hoc tests revealed that familiarity ratings in the latter sample were significantly lower for the category of toys, as compared to the category of kitchen utensils and of office supplies; familiarity for the women’s accessories was also significantly lower relative to office supplies (see Table 5 for descriptive values by category).

**Table 5:** Mean (and Standard Deviation) for name agreement scores (%NA and *H*) and familiarity ratings for each category and each sample.

	%NA			H			Familiarity		
	American^a^	Port.^a^	Port.^b^	American^a^	Port.^a^	Port.^b^	American^a^	Port.^a^	Port.^b^
**Women’s Accessories**	78.3 (21.0)	83.8 (14.9)	79.4 (18.4)	0.61 (0.62)	0.65 (0.59)	0.70 (0.64)	4.06 (0.52)	4.39 (0.30)	4.25 (0.38)
**Fruits**	72.2 (34.2)	88.9 (16.0)	89.1 (15.9)	0.66 (0.81)	0.33 (0.52)	0.34 (0.51)	4.07 (0.95)	4.62 (0.44)	4.59 (0.40)
**Kitchen utensils**	72.9 (25.2)	74.7 (23.9)	74.7 (23.5)	0.80 (0.78)	0.79 (0.73)	0.75 (0.70)	4.29 (0.66)	4.67 (0.25)	4.64 (0.26)
**Office Supplies**	70.5 (30.4)	86.3 (18.2)	88.5 (15.7)	0.75 (0.86)	0.45 (0.59)	0.40 (0.55)	4.31 (0.80)	4.73 (0.20)	4.68 (0.19)
**Toys**	87.9 (18.3)	88.4 (14.3)	91.3 (10.8)	0.31 (0.45)	0.32 (0.38)	0.28 (0.34)	4.45 (0.44)	4.33 (0.39)	4.33 (0.32)
**Vegetables**	66.6 (30.2)	83.3 (17.4)	81.5 (17.8)	0.90 (0.89)	0.50 (0.54)	0.58 (0.60)	3.99 (0.80)	4.52 (0.43)	4.51 (0.44)

American = North American sample; Port. = Portuguese sample

a = clean condition

b = sauce condition

Comparisons between the two samples should, however, be taken with care given some sociodemographic differences between them. For example, the North American Sample was significantly younger than the Portuguese sample, *t*(246) = 10.59, *p* < .001, Cohen’s *d* = 1.453. Also, the former sample was composed solely of undergraduate students whereas the Portuguese sample included participants with different occupations.

### Data from the Portuguese sample (sauce condition)

Each of the 126 stimuli from the sauce condition was, on average, rated by 60.6 (*SD* = 1.1) native European Portuguese participants. The percentage of participants who gave the modal name (%NA) was 84.3% (*SD* = 17.9%; range: 33.3% - 100%) and the *H* value was 0.50 (*SD* = 0.58; range: 0–2.22) (see Table 1; for an in-depth analysis see Tables 2 and 3). Again, %NA and H were highly negatively correlated, *r*_*s*_ = -.948, *p* < .001.

Only 4.4% of the data represent naming failures, with most referring to DKN responses (see Table 4). From those participants who named the stimuli (i.e., excluding naming failures), 87.8% provided the modal name, 7.6% provided a correct nonmodal name and 4.6% gave an incorrect nonmodal name. Nonmodal names were similar to those described previously for the clean condition from the Portuguese sample.

Participants were in general very familiar with the stimuli (*M* = 4.49, *SD* = 0.37; scale: 1–5). Familiarity was highly positively correlated with %NA (*r*_*s*_ = .589, *p* < .001), and negatively correlated with the *H* value (*r*_*s*_ = -.501, *p* < .001).

### Comparing data from the clean and the sauce condition

The last presented data allowed us to explore if the norming information obtained for the stimuli from the clean condition would be representative of the data one would obtain in the other presentation conditions. In what follows, we compared the data obtained for the stimuli from the clean condition with those from the sauce condition to help answer this question. Overall, the naming results obtained for the stimuli from the sauce condition were similar to those obtained when the objects were held by clean hands (see Table 1).

A mixed ANOVA with stimulus condition (clean vs. sauce) and category as factors revealed no significant main effect of stimuli condition on each of the naming variables (%NA and *H* value; both *Fs* < 1). These data provide initial evidence that the naming norms obtained for stimuli on clean hands can be generalized to the other conditions. For both naming metrics, though, we obtained a significant main effect of category, *F(*5, 120) = 2.31, *MSE* = 0.13, *p* = .048, η_*p*_^2^ = .088, and *F(*5, 120) = 2.44, *MSE* = 1.48, *p* = .039, η_*p*_^2^ = .092, for %NA and *H* value, respectively. However, the post-hoc analysis revealed no significant differences in the comparisons between the pairs of categories. The Condition X Category interaction was significant for the %NA, *F(*5, 120) = 2.73, *MSE* = 0.01, *p* = .022, η_*p*_^2^ = .102, but not for the *H* value, *F(*5, 120) < 1. When we compared the %NA among categories in each sample, no significant effect was obtained in the clean condition, *F*(5,120) = 1.72, *p* = .135, but it was reliable on the sauce condition, *F(*5, 120) = 2.96, *MSE* = 0.09, *p* = .015, η_*p*_^2^ = .110. Gabriel's post-hoc analysis revealed lower %NA (i.e., fewer participants giving the modal name) for stimuli from the category of kitchen utensils compared to those from the category of toys.

Furthermore, neither the main effects of stimuli condition or of category, nor the interaction between these variables, reached significant values on each of the naming failures (DKO, DKN and TOT; highest F value for the main effect of sample for the TOT: *F*(1, 120) = 3.47, *p* = .065).

Regarding familiarity, there was, however, a significant effect of stimulus condition, *F(*1, 120) = 8.68, *MSE* = 0.12, *p* = .004, η_*p*_^2^ = .067, with participants reporting less familiarity when the objects were held by hands covered with sauce as compared to when they were being held by clean hands. The main effect of category was also significant, *F*(5, 120) = 5.24, *MSE* = 1.18, *p* < .001, η_*p*_^2^ = .179, with participants reporting being less familiar with the objects from the women’s accessories and toys as compared to office supplies and kitchen utensils. These differences resemble those reported when the objects were held by clean hands (see above). The interaction between the two variables was not significant, *F(*5, 120) = 2.18, *MSE* = 0.03, *p* = .061, η_*p*_^2^ = .083.

## Interim conclusion

In conclusion, the results revealed high name agreement and familiarity in both the North American and the Portuguese samples for objects on clean hands, even though the Portuguese sample was more accurate in naming the stimuli and reported being more familiar with them. The data from an additional Portuguese sample for the objects held by hands covered with sauce resembled those collected with objects on clean hands.

## Study 2: Arousal, disgust and emotional valence

Study 2 had two primary goals. The first goal was to evaluate whether participants' affective ratings of images differed depending on the condition in which the object was photographed, i.e., to analyze if the intensity of the emotional activation promoted by the stimuli varied according to the substance covering the hands. To that end, participants rated images either from the chocolate, sauce or mud condition. No context was provided for this task, that is, participants were not given any description about the nature of the pictures they were going to assess.

Secondly, we wanted to determine whether the activation of the affective dimensions afforded by the stimuli would differ depending on the encoding context in which objects were framed. Thus, photographs from the chocolate condition were described in the context of a disease situation context (hands described as being covered by diarrhea), or in a non-disease context (hands described as being covered by chocolate spread). The ratings obtained in these two contexts were also compared with those obtained previously when no context framing was presented. We expected that the participants would rate the various affective dimensions as more negative when framed in the disease than in the non-disease context.

In all procedures, the “dirty” hands were intermixed with images from the clean condition which we expected would afford lower emotional activation than hands covered with substances. Ratings for all of these cases were collected across three dimensions—arousal, disgust and emotional valence, via an online questionnaire.

## Method

### Participants

A total of 970 participants took part in the study. Given the high dropout rates of online questionnaires, data were considered whenever at least half of the stimuli had been rated. Five hundred and fifty-eight participants fulfilled this criterion (translating into a dropout rate of 42.5%). Participants younger than 18 years (*n* = 1) or with a nationality other than Portuguese (*n* = 19) were also excluded from the analysis. The final sample included 538 native European Portuguese speakers (women = 365; 68.61%), aged between 18 and 74 years (*M* = 35.90, *SD* = 13.15). From this sample, 313 participants (*M*_*age*_ = 35.46, *SD* = 12.94) provided the ratings for the objects with no context described. A total of 111 participants (*M*_*age*_ = 36.40, *SD* = 12.91) provided this same information for the objects from the chocolate condition described in the disease context and 114 participants (*M*_*age*_ = 36.64, *SD* = 14.01) for the same stimuli described in the non-disease condition. No compensation was offered to participate in the study. All participants responded in a voluntary manner and provided initial consent.

### Materials

In this task, the 126 frontal-view stimuli from the clean, mud, sauce and chocolate conditions were used. However, each participant provided ratings to a set of 63 stimuli previously created in a pseudo-random manner so that: (1) a similar number of objects from each of the six categories was presented, and (2) a similar number of objects from the clean condition and from one of the “dirty” conditions was presented. This procedure created 2 sets of stimuli for each presentation condition. Within each set, half of the stimuli were presented in clean-hands and the other half in the hands covered with a substance; each object was only presented once (either in the clean or in the substance condition) to a given participant. Counterbalancing versions of the questionnaire ensured that a given object would be rated a similar number of times in the clean condition and in each of the substances conditions. In all cases, the “dirty” hands were intermixed with images from the clean condition. Order of presentation of the stimuli was randomly determined for each participant.

### Procedure

The questionnaires were administered via the World Wide Web using the *Qualtrics survey software* (Qualtrics Labs Inc., Provo, UT). An electronic mail was sent to several entities (e.g., universities, professional schools and other companies across Portugal); this included a brief description of the study along with a unique electronic link to access the questionnaire. The opening page of the questionnaire consisted of further information about the study along with an informed consent request. If no consent was given, participants were thanked for their interest and the program ended; otherwise, the program moved on.

Demographic information including sex, age, and nationality were first collected. Participants were then randomly assigned to one of the three contexts (disease context, non-disease context, or no context) and to one of the counterbalancing versions of the questionnaire. The context and version of the experiment to which subjects were assigned was randomly selected by *Qualtrics*. For the assessment of the stimuli varying the substances covering the hands (mud, sauce, or chocolate condition) and with no context provided, participants were presented with the following instructions:

*No context*: “In this task, you will see pictures of objects being held by hands. You will be asked to evaluate each image in several dimensions. Each question will be followed by its response options; select your answer by clicking on the number corresponding to your choice.”

In the ‘disease’ and ‘non-disease’ encoding contexts, participants rated the stimuli presenting the hands covered with chocolate but these were framed in a disease or a non-disease context as follows:

Disease context: “In this task, you will see pictures of objects that have been touched by different people. One of these people is sick with a highly contagious gastrointestinal infection and is having severe and frequent episodes of diarrhea. Sometimes he/she cannot reach the toilet on time and gets diarrhea on his/her hands while handling objects. The other person is healthy and is handling objects with clean hands. Throughout the experiment, you will see pictures of objects held by hands covered with diarrhea or clean hands.”

Non-disease context: “In this task, you will see pictures of objects that have been touched by different people. One of these people has been making cakes and his/her hands are covered in chocolate spread. Sometimes he/she cannot find time to clean his/her hands and has chocolate spread on them while handling objects. The other person is handling objects with clean hands. Throughout the experiment, you will see pictures of objects held by hands covered with chocolate spread or clean hands.”

In all cases, each stimulus was presented one at a time on the computer screen; questions and their corresponding response options were shown below each image. Participants were asked to use a 9-point Likert scale to indicate (a) how calm or excited each picture made them feel (i.e. arousal: 1-very calm, 9-very excited); (b) how disgusted each picture made them feel (i.e. disgust: 1-not at all disgusted, 9-very disgusted) and, (c) how negative or positive was each image for them (emotional valence: 1-very negative, 9-very positive). A SAM (Self-Assessment Manikin) scale (Lang, 1980) was used to measure valence and arousal. Responses were provided by clicking on the response of their choice with the computer mouse. The tasks were self-paced but participants were instructed to respond quickly and to rely on their “gut instinct”. After responding to all questions participants hit a “next” button which led to the presentation of the next stimulus. The task lasted approximately 15 minutes.

### Statistical analysis

To test if the participants' ratings differed depending on the substance covering the hands, a two-way univariate ANOVA was employed, with ‘image condition’ (hand covered with chocolate, sauce, and mud, generally named as “dirty hands”) and ‘state of the hands’ (dirty and clean) as between-subjects factors. When interactions were significant, follow-up one-way ANOVAs and post hoc Bonferroni tests were performed separately for each ‘state of the hands’ to pinpoint the source of the interaction.

To test if participants' ratings differed depending on the encoding context manipulation, a mixed ANOVA was performed with ‘encoding context’ (disease context, non-disease context and no context) as the within-subject variable and the ‘state of the hands’ (dirty and clean) as the between-subjects variable. When interactions were found to be significant, follow-up repeated-measures ANOVAs were additionally carried out separately for each ‘state of the hands’. When the sphericity assumption was not met (as evaluated using Mauchly's test), a Greenhouse-Geisser correction was applied. The level of statistical significance was set at .05 (two-tailed) for all reported analysis.

## Results and discussion

Across all of the conditions employed here, each image was rated by approximately 25 participants (*SD* = 2.50). The average number of responses obtained for the images separated by image condition and context is presented in Tables 6 and 7; the statistical results of the overall ANOVAs are also reported in these Tables. The ratings provided for each stimulus, in each encoding context, are available as S4 Appendix (available at https://osf.io/xn2u9/).

**Table 6:** Mean number of responses (and SD) obtained per image condition and mean ratings (and SD) of arousal, disgust and valence in each image condition when no context was presented.

	Chocolate *vs*. Clean condition		Sauce *vs*. Clean condition		Mud *vs*. Clean condition		
Mean Number	23.92 (1.07)		25.67 (3.41)		23.08 (1.12)		
	chocolate	clean	sauce	clean	mud	clean	Statistics
**AROUSAL**	4.68(0.45)	2.75 (0.42)	4.00 (0.34)	2.40 (0.36)	3.41 (0.68)	2.44 (0.53)	**ME subst:** *F*(2,750) = 178.03, *MSE* = 40.7, η_*p*_^2^ = .322*** ME SH: *F*(1,750) = 1866.76, *MSE* = 426.8, η_*p*_^2^ = .713*** Interact: *F*(2,750) = 65.99, *MSE* = 15.1, η_*p*_^2^ = .150***
**DISGUST**	4.72 (0.42)	1.53 (0.32)	4.34 (0.44)	1.42 (0.21)	3.05 (0.56)	1.25 (0.19)	**ME subst:** *F*(2,750) = 449.64, *MSE* = 64.9, η_*p*_^2^ = .545*** ME SH: *F*(1,750) = 9098.02, *MSE* = 1313.7, η_*p*_^2^ = .924*** Interact: *F*(2,750) = 238.63, *MSE* = 34.5, η_*p*_^2^ = .389***
**VALENCE**	3.79 (0.31)	6.21 (0.59)	4.08 (0.37)	6.35 (0.50)	4.75 (0.56)	6.25 (0.55)	**ME subst:** *F*(2,750) = 67.13, *MSE* = 16.1, η_*p*_^2^ = .152*** ME SH: *F*(1,750) = 3366.51, *MSE* = 808.5, η_*p*_^2^ = .818*** Interact: *F*(2,750) = 64.64, *MSE* = 15.5, η_*p*_^2^ = .147***

Note: Mean number: Mean number of responses; ME subst: Main effect of substance (mud, sauce, chocolate); ME SH: Main effect of state of hands (clean, dirty); Interact: interaction between variables

*** *p* < .001

**Table 7:** Mean number of responses (and SD) obtained per image and mean ratings (and SD) of arousal, disgust and valence in each encoding context.

	Disease context		Non-disease context		No context		
Mean Number	26.03 (2.26)		26.56 (1.87)		23.92 (1.07)		
	dirty	clean	dirty	clean	dirty	clean	Statistics
**AROUSAL**	5.42 (0.52)	2.00 (0.31)	4.55 (0.72)	2.26 (0.39)	4.68 (0.45)	2.75 (0.42)	**ME context:** *F*(1.7, 414.8) = 30.93, *MSE* = 9.6, η_*p*_^2^ = .110*** ME SH: *F*(1,250) = 6270.44, *MSE* = 1229.1, η_*p*_^2^ = .962*** Interact: *F*(1.7, 414.8) = 148.86, *MSE* = 46.2, η_*p*_^2^ = .373***
**DISGUST**	6.22 (0.34)	1.45 (0.25)	4.72 (0.43)	1.45 (0.23)	4.72 (0.42)	1.53 (0.32)	**ME context:** *F*(2,500) = 407.92, *MSE* = 45.2, η_*p*_^2^ = .620*** ME SH: *F*(1,250) = 21517.79, *MSE* = 2651.0, η_*p*_^2^ = .989*** Interact: *F*(2,500) = 452.82, *MSE* = 50.2, η_*p*_^2^ = .644***
**VALENCE**	3.00 (0.34)	6.95 (0.41)	3.74 (0.38)	6.68 (0.58)	3.79 (0.31)	6.21 (0.59)	**ME context:** *F*(2,500) = 36.87, *MSE* = 4.24, η_*p*_^2^ = .129*** ME SH: *F*(1,250) = 4834.12, *MSE* = 1820.6, η_*p*_^2^ = .951*** Interact: *F*(2,500) = 326.49, *MSE* = 37.6, η_*p*_^2^ = .566***

Note: Mean number: Mean number of responses; ME context: Main effect of context (disease, no-disease, no-context); ME SH: Main effect of State of hands (clean vs. dirty); Interact: interaction between variables

*** p ***<* .*001***

### Emotional activation as a function of the substance covering the hands (no context)

For Arousal, both the main effects of substance covering the hands and of state of the hands were statistically significant. The former indicates that the different substances induced different levels of arousal and the latter reflects that the dirty hands stimuli were considered more arousing than the clean hands stimuli. However, the interaction between these two variables was also significant (see Table 6). When analyzing the data separately by *state of the hands*, a significant effect was found for the dirty hands, *F*(2, 375) = 197.87, *MSE* = 51.1, *p* < .001, η^2^ = .514. Follow-up analysis showed that the stimuli from the chocolate condition elicited the highest level of arousal, followed by the stimuli from the sauce condition and then by those from the mud condition, which elicited the lowest ratings. Arousal ratings for objects on clean hands were also influenced by the type of dirty hands presented concurrently, *F*(2, 375) = 23.53, *MSE* = 4.7, *p* < .001, η^2^ = .112; when these were presented along with images from the chocolate condition they were rated as being more arousing than when presented with images from the sauce and the mud conditions (see Table 6).

Disgust ratings also differed considerably depending on the substance covering the dirty hands, as shown by the significant main effects of substance, and of state of the hands (the dirty hands stimuli were rated as more disgusting than the clean hands stimuli). These main effects were also qualified by a significant interaction between the two factors (see Table 6). Subsequent analysis looking into each variable revealed significant effects both within the dirty hands and the clean hands, *F*(2, 375) = 424.56, *MSE* = 96.9, *p* < .001, η^2^ = .694, and *F*(2, 375) = 41.09, *MSE* = 2.5, *p* < .001, η^2^ = .180, respectively). These results reflect the fact that participants reported considerably and significantly higher disgust ratings for both the objects on dirty hands and objects on clean hands when the hands were covered with chocolate as compared to the sauce and mud substances; a slightly higher disgust was also obtained when the hands were dirty with sauce than with mud (see Table 6).

Finally, Valence ratings also varied significantly according to the substance covering the hands and the state of the hands (when covered with a substance, the stimuli were considered more negatively valenced than when they were clean). A significant interaction was also obtained for this variable. Follow-up analysis revealed significant differences among objects on dirty hands but not among objects on clean hands, *F*(2, 375) = 172.69, *MSE* = 31.0, *p* < .001, η^2^ = .479, and *F*(2, 375) = 2.28, *p* = .104, respectively, with more negative valence ratings being assigned for the dirty hands from the chocolate condition, followed by those from the sauce condition and, finally, by those from the mud condition (see Table 6).

### Emotional activation depending on the encoding context

In these analyses we compared the ratings obtained for the objects being held by hands covered with chocolate or clean hands when these were framed in three conditions: disease context, no-disease context and no-context; the data from this last condition were the same as those considered in the previous set of analysis.

Our results revealed that the manipulation of the encoding context can differently prompt Arousal, as shown by significant main effects of context and of state of the hands, but also by a significant interaction between the two. When the influence of context was analyzed for the dirty hands, a significant effect was obtained, *F*(1.3, 164.6) = 71.07, *MSE* = 42.3, *p* < .001, η_*p*_^2^ = .362. Follow-up analysis revealed that participants became more aroused when objects on dirty hands were described in the disease context as compared to when the same objects were either considered in a non-disease context or when no type of framing is given (the difference between the latter two was not significant). The contextual framework seems, likewise, even to influence the arousal level of objects on clean hands (although these images were described in the same way in the disease and the non-disease contexts), *F*(1.7, 215.2) = 150.11, *MSE* = 21.4, *p* < .001, η_*p*_^2^ = .546. Participants felt most aroused when viewing objects on clean hands with no context was provided, less aroused when viewing the same images in the non-disease context, and least aroused when viewing the images in the disease context (see Table 7). Note that this pattern is the opposite of that obtained for the dirty hands which might suggest some form of contrast effect between the two types of stimuli.

Similarly for Disgust, the results yielded significant main effects of context and state of the hands. Additionally, a significant interaction between the two variables was found. The analyses that followed the discovery of a significant main effect of context for the dirty hands, *F*(2, 250) = 647.17, *MSE* = 95.1, *p* < .001, η_*p*_^2^ = .838, revealed that the participants reported feeling significantly more disgusted when a disease context was described than when a non-disease context or no context was provided at all (the difference between the latter two was not significant). Regarding the same analysis for the significant main effect obtained for the clean hands, *F*(1.6, 195.4) = 3.79, *MSE* = 0.36, *p* = .034, η_*p*_^2^ = .029, the results revealed that, on average, disgust ratings for objects on clean hands were relatively higher when no context was given compared to a no-disease context; the later did not significantly differ from the disease context (see Table 7).

Finally, for emotional Valence, there were significant main effects of context and state of the hands, which were qualified by an interaction between the two variables. A significant effect of context was obtained for the dirty hands, *F*(1.8, 235.2) = 217.39, *MSE* = 26.6, *p* < .001, η_*p*_^2^ = .635, with stimuli being rated as more negatively valenced when framed in a disease context compared to when they were either framed in a no-disease context or when no context was given. The valence ratings for the objects on clean hands were also significantly affected by the context framing, *F*(1.9, 235.2) = 147.9, *MSE* = 18.6, *p* < .001, η_*p*_^2^ = .542; the follow-up analyses revealed that these objects prompted a more positively valenced emotion when presented in the disease context, followed by lower valence ratings in the no-disease context and even lower ratings in the no context condition (see Table 7).

## Interim conclusion

In sum, our data regarding the emotional activation as a function of the substance covering the hands when no context was provided revealed that the emotional responses differed by substance. Specifically, the images from the chocolate condition were rated as being the most arousing, disgusting and negatively valenced, followed by the sauce and then by the mud condition. These results seem to be consistent with the Oaten and collaborator’s [34] proposal that disgust is likely to be evoked in proportion to the infection load (i.e., the disease risk) of a stimulus. The authors suggested that “one could potentially calculate the number of pathogen species that each disgust elicitor carried and correlate this to the degree of reported disgust that each cue evokes” (p. 307). In fact, 1 g of feces contains an estimated 10^12^ viral particles whereas 1 ml of vomit contains around 10^7^ [46]. Therefore, the higher the likelihood of contamination, the stronger the disgust reaction.

The ratings for the clean hands stimuli which were rated when intermixed with the various dirty hands differed for the dimensions of arousal and disgust but not for valence. Regarding the first two, the pattern of results followed that obtained for the dirty hands that accompanied them: they were rated as most arousing and disgusting when intermixed with the hands from the chocolate condition. Such data suggest some form of emotional contagion from the dirty to the clean hands stimuli when no context is provided.

Regarding the influence of the contextual influence on emotion overall, our results revealed that framing the exact same stimuli in different ways (e.g., describing the same dirty hands as covered with diarrhea or chocolate, or providing no description at all) influenced the intensity of the participants’ emotional responses in the various affective dimensions evaluated here. When the objects were held by the dirty hands described as covered with diarrhea (disease context), they were consistently rated as more arousing, disgusting and negatively valenced, as compared to when the hands were described as being covered with chocolate (non-disease context) or when no context was provided. The contextual framework seems, likewise, to impact the emotional ratings of the clean hands stimuli when these were presented in the same context framings and intermixed with the dirty hands stimuli. However, here, the pattern of results followed the opposite tendency of that obtained for the dirty hands (clean hand stimuli were rated as least arousing, disgusting and most positively valenced in the disease condition) as if a contrast effect was occurring.

## General conclusion

We developed and validated a new database of stimuli that comprises high-quality color photographs of everyday objects recorded under two camera viewpoints and five presentation conditions. Even though many image datasets exist, to the best of our knowledge, none provides photographs of the exact same stimulus in different presentation conditions. The variation of the context in which objects are presented in this set of stimuli (e.g., clean hands condition, mud condition) affords new forms of manipulation while keeping the stimuli of interest (i.e., the object itself) the same, thus minimizing item-selection concerns of the type often found in research studies. Furthermore, the objects can be organized into six different categories providing an additional organizational dimension that can be of interest to researchers. Additionally, by providing two camera viewpoints, we amplify the spectrum of different scenarios that can be created, including scenarios that involve different forms of social interaction (e.g., receiving vs. giving away). There are, however, some limitations in the application of such conceptualizations that we should point out. For example, skin color typically differs from person to person: whereas Caucasians could easily accept the displayed hands as their own, the same is unlikely to happen for non-white participants. Such limitation, however, can be seen as an opportunity for further development of the database. For example, in the future, the database could be complemented with additional photographs using hands with varying skin colors. Alternatively, the already existing photos could be edited to change the skin tones according to the researchers’ goals.

This study showed high name agreement and a relatively high degree of familiarity in both North American and Portuguese samples, although some cross-cultural differences were found. By providing the data from these two samples, one can ensure the selection of stimuli that can be equally named and are equally familiar between the two groups, allowing cross-cultural studies to be conducted. The North American sample had some difficulty in naming some of the items, particularly from the fruits and vegetables categories. This could be due to the fact that the stimuli were picked in Portugal and are not necessarily common in the USA. Given this potential for idiosyncrasies across countries future studies should aim to collect normative data in other countries and cultures expanding the potential for this database to be used in cross-cultural experiments.

The results from study 2 confirmed that the different presentation conditions of our stimuli (via the different substances covering the dirty hands) can induce different emotional states. In particular, the images from the chocolate condition were considered the most arousing, disgusting, and negatively valenced, followed by the sauce, and then by the mud condition. Furthermore, we showed that the exact same stimuli can be used to afford different emotional states by simply framing them in different contexts: when the objects from the chocolate condition were described as being covered with diarrhea (disease context) they were consistently rated as more arousing, disgusting and negatively valenced as compared to when they were described as being covered with chocolate (non-disease context) or when no context was provided. A similar variety of contexts could be created for photographs from the other conditions; for example, photographs from the sauce condition could be described as belonging to a vomit or to a pasta sauce condition. Future studies should collect additional information in order to confirm that the participants’ perception of the stimuli follows our framing (i.e., they believe the hands are covered with chocolate or diarrhea, for example). Nonetheless, the subjective emotional ratings we obtained seem to suggest that participants did believe in the descriptions accompanying the pictures. These initial data reassure researchers that they can keep the object of interest the same across their experiments (thus minimizing the influence of item-specific characteristics), differing only in the emotional reactions elicited by either manipulating the hands condition (e.g., hands covered with chocolate or pasta sauce) or the encoding context (i.e., the cover story provided with the stimuli). It would also be interesting for upcoming studies to complement the existing subjective data with more objective indicators of emotional reaction (e.g., physiological data).

Hence, the database comprises a suitable set of pictures of everyday objects that can be used by a large number of researchers from different knowledge domains and with various research goals. We provide subjective data regarding the variables of arousal, disgust and emotional valence but there are many other cognitive and psycholinguistic variables of potential interest to researchers that could be collected in future studies (e.g., age of acquisition, familiarity, typicality, manipulability, pleasantness or naming latency). Other subjective and more objective variables regarding the perceptive aspects of the stimuli could also complement this database, such as subjective and objective indicators of visual complexity.

This database is freely available for scientific purposes after submitting formal request for research use via the database website: https://sites.google.com/view/adaptive-memory-lab/data-databases. By making our norming data available through the Open Science Framework (OSF) project we ensure their permanent availability. We aim to maintain our dataset website and OSF project updated with further developments of the database and with references of studies that have used it. Moreover, if requested by other researchers, we would be happy to also include more specific information about further norming studies on our website. We look forward to reference exciting and innovative research using our stimuli.

## Supporting information

S1 Appendix List of pictures available in each condition and viewpoint. (XLSX)

S2 Appendix Indexes related to the naming task and familiarity ratings obtained for each stimulus and in each sample. (XLSX)

S3 Appendix Responses coded as modal names, the different alternative names provided, along with their corresponding frequencies and percentages of occurrence. (XLSX)

S4 Appendix Arousal, disgust, and valence ratings provided for each stimulus, in each encoding context. (XLSX)
